# Special Issue: Emerging Topics in Metal Complexes: Pharmacological Activity, 2nd Edition

**DOI:** 10.3390/ijms262412165

**Published:** 2025-12-18

**Authors:** Agnieszka Ścibior, Manuel Aureliano, Juan Llopis

**Affiliations:** 1 Laboratory of Oxidative Stress, Department of Biomedicine and Environmental Research, The John Paul II Catholic University of Lublin, 20-708 Lublin, Poland; 2 Faculdade de Ciências e Tecnologia (FCT), Campus de Gambelas, Universidade do Algarve, 8005-139 Faro, Portugal; 3 Centro de Ciências do Mar do Algarve (CCMar/CIMAR LA), Campus de Gambelas, Universidade do Algarve, 8005-139 Faro, Portugal; 4 Department of Physiology, Institute of Nutrition and Food Technology, Biomedial Research Center, Institute of Biosanitary Research, University of Granada, 18071 Granada, Spain; jllopis@ugr.es

## 1. Introduction and Scope

**Figure 1:**
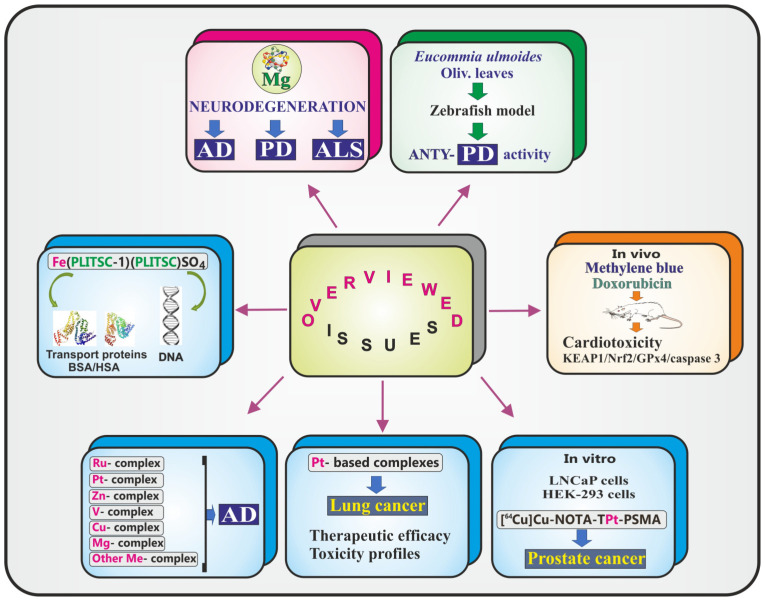
Graphical summary of the included issues: studies on metals (red); studies involving metal complexes (blue); studies on extracts of plant origin (green); and studies investigating drugs (orange).

This Special Issue (SI), titled “Emerging Topics in Metal Complexes: Pharmacological Activity, 2nd Edition”, includes reports updating our knowledge about magnesium (Mg) and neurodegeneration, various metal-containing complexes in terms of their potential therapeutic applications (i.e., ruthenium (Ru), platinum (Pt), zinc (Zn), vanadium (V), copper (Cu), magnesium (Mg), and other metal complexes and their mechanisms in treating Alzheimer’s disease (AD)), and the efficacy of platinum (Pt)-based complexes in the treatment of lung cancer. It also includes research into a promising radiotheranostic agent against prostate cancer and a novel Fe(III)-pyridoxal-derivative complex with serum proteins and DNA binding activities. In addition, this SI provides valuable findings on the efficacy of methylene blue (MB) in protecting the heart from doxorubicin (Dox)-induced damage and addresses the identification of key active constituents in *Eucommia ulmoides* Oliv. leaves (EUOL) against Parkinson’s disease (PD) and their underlying mechanisms. The scientific articles making up this SI, i.e., three review articles and four original papers (seven in total), have garnered a total of 16 citations and 17,528 views, indicating an average of 2 citations and 2,504 views per publication (29 November 2025). Figure 1 summarizes the issues included in this SI.

## 2. Contributions

### 2.1. Metals

The first paper published in this Special Issue, by Ścibior et al. [contribution 1], titled “Magnesium (Mg) and neurodegeneration: a comprehensive overview of studies on Mg levels in biological specimens in humans affected some neurodegenerative disorders with an update on therapy and clinical trials supplemented with selected animal studies”, aims to provide thorough knowledge on a possible association between Mg levels and neurodegenerative disorders such as Alzheimer’s disease (AD), Parkinson’s disease (PD), and Amyotrophic lateral sclerosis (ALS) in humans. Hence, this review compiles data on the concentrations of Mg in blood, cerebrospinal fluid (CSF), urine, and hair from subjects with AD, PD, and ALS to detect possible variations in the levels of this essential element in the biological specimens of people with neurodegenerative illnesses. Additionally, the review article provides basic information about Mg^2+^ and its biological function; describes the role of this mineral in the brain and the symptoms of its deficiency, mainly focusing on the nervous system; summarizes data on the neuroprotective mechanisms of Mg^2+^ in AD, PD, and ALS; and collects information about the neuroprotective efficacy of this bioelement following experimental brain injury in animals. Moreover, this work also provides a summary of studies on animals and humans on the neuroprotective efficacy of Mg in the context of cognitive function, and gives an overview of the therapeutic effects of Mg in humans with AD, PD, and ALS. Finally, it compiles information on the use of Mg in clinical trials on humans with AD, PD, and ALS. The data provided in this review indicate that Mg, due to its neuroprotective, antioxidant, anti-inflammatory, and mitochondrial-supportive properties, could be a potential therapeutic agent for AD, PD, and ALS. However, as the authors emphasize, further epidemiological studies with standardized methods of dietary assessment and Mg measurement are necessary to be able to recognize its exact role in neurodegenerative disorders. Moreover, they draw attention to the need to conduct extensive, well-designed clinical trials to establish definitive therapeutic protocols and optimal dosages, and to ensure the long-term safety of the supplementation of this mineral in AD, PD, and ALS patients. Recent research from Ścibior’s group includes the pharmacological potential of vanadium and the mechanisms underlying its anti-viral, anti-bacterial, anti-parasitic, anti-fungal, anti-cancer, anti-diabetic, anti-hypercholesterolemic, cardioprotective, and neuroprotective activity, as well as its mechanisms of appetite regulation, with regard to the possibility of using this element in the treatment of obesity [1].

### 2.2. Metal Complexes

One of the review articles included in this SI focuses on certain metal complexes, i.e., ruthenium (Ru), platinum (Pt), zinc (Zn), vanadium (V), copper (Cu), magnesium (Mg), and other metal complexes and their mechanisms of action in treating Alzheimer’s disease (AD) [contribution 2], which is a chronic, progressive neurodegenerative disorder characterized by cognitive decline, memory loss, and impaired reasoning [2]. As we know, metal dyshomeostasis disrupts normal ion balance in the brain and plays a critical role in AD by influencing beta-amyloid (Aβ) aggregation, oxidative damage, and neural degeneration [3]. In turn, metal complexes can restore metal ion homeostasis, disrupt harmful metal–Aβ interactions, and scavenge free radicals, thereby slowing or reversing AD progression. However, as stressed by the authors, the dosage of metal complexes should be controlled to prevent damage to neurons. Additionally, improving the stability of metal complexes to ensure their effectiveness and mechanistic understanding has been highlighted as being essential for optimizing the use of these compounds as treatments. To summarize, metal complexes are a promising avenue for AD treatment aiming to normalize metal homeostasis and mitigate neurotoxicity. They represent a promising direction for the development of new drugs that could break down pathological Aβ peptides, inhibiting the formation of aggregates. Besides metal complexes, reviews on polyoxometalates (POMs) focusing on AD have more than doubled over the past two years, reflecting a marked increase in the research interest in these fields since the first study was produced in 2011 [4,5,6].

Another review paper comprehensively discusses the development of platinum (Pt) compounds used in the treatment of lung cancer, with particular emphasis on three generations of Pt drugs, namely cisplatin (first generation), carboplatin (second generation), and oxaliplatin (third generation) [contribution 3]. The article highlights the challenges of Pt therapy, such as systemic toxicity, development of resistance, and side effects, that limit the use of these drugs in large doses and for extended periods of time. Therefore, in response to these issues, it is necessary to develop newer generations of Pt drugs and investigate experimental compounds with a better therapeutic profile and fewer side effects. To summarize, Pt complexes still make up the foundation of lung cancer therapy, and the latest research focuses on optimizing their efficacy, minimizing side effects, and overcoming treatment resistance, which enables the development of so-called next-generation drugs and combination therapies.

This SI also provides new information about a novel radiotheranostic agent, i.e., [^64^Cu]-NOTA-TP-PSMA, which was generated by conjugating a prostate-specific membrane antigen (PSMA) ligand with a ^64^Cu-radiolabeled terpyridine-platinum (TP) complex. Talebian et al. [contribution 4] investigated the in vitro selectivity of this complex and its internalization, in addition to its cytotoxicity on PSMA-positive LNCaP prostate cell line and non-malignant human embryonic kidney (HEK-293) cells. The study demonstrated that [^64^Cu]-NOTA-TP-PSMA showed enhanced specificity, uptake, internalization, retention, nuclear localization in LNCaP cells, and cytotoxic potency relative to the monomeric radioligands. The authors stressed the need for further studies to clarify the radiobiological mechanisms underlying the increased in vitro cytotoxicity of [^64^Cu]-NOTA-TP-PSMA and to assess its potential as a targeted radioligand for the imaging and therapy of prostate cancer, which is the second most common cancer among men worldwide [7].

Moreover, the current SI includes an original article focusing on the crystallographic structure, theoretical analysis, and protein/DNA binding activity of the bis-ligand-iron(III) complex, which, as stressed by the authors, is particularly interesting due to the presence of two differently protonated pyridoxal-S-methyl-isothiosemicarbazone (PLITSC) ligands, i.e., ([Fe(PLITSC-1)(PLITSC)]SO_4_) [contribution 5]. This study details the preparation of the iron(III) complex with PLITSC ligands, emphasizing the importance of ligand protonation in dictating the structure and stability of the complex, and deduces the geometric configurations of the complex, correlating crystallographic studies with theoretical analysis. In turn, the analysis of the biological activity of ([Fe(PLITSC-1)(PLITSC)]SO_4_), assessed based on its binding affinity towards transport proteins, such as human serum albumin (HSA), bovine serum albumin (BSA), and DNA, showed that the [Fe(PLITSC-1)(PLITSC)]^2+^ complex binds to the active positions of proteins and to DNA. The authors demonstrated a notable interaction influenced by the protonation state of the ligands. The binding affinity of [Fe(PLITSC-1)(PLITSC)]^2+^ towards BSA was observed to range between −31.0 and −34.9 kJ mol^−1^ (27–37 °C), while in the case of HSA, it was noted to be between −33.0 and −36.4 kJ mol^−1^ (27–37 °C). The values obtained from molecular docking simulations were −28.8 (BSA) and −30.3 (HSA) kJ mol^−1^. As far as DNA is concerned, the binding affinity of the [Fe(PLITSC-1)(PLITSC)]^2+^ complex to the DNA structure was found to be −25.0 kJ mol^−1^, and the calculated binding energy was noted to be −27.6 kJ mol^−1^. These results suggest potential applications of the [Fe(PLITSC-1)(PLITSC)]^2+^ complex in biomedical fields, particularly in designing therapeutics that target protein and DNA structures. To summarize, the findings presented in this work indicate the way towards the development of new metal-based complexes with tailored binding properties for applications in biochemistry and medicinal chemistry and contribute to the elucidation of the impact of structural differences in metal complexes on their interactions with biological macromolecules, paving the way for future studies on similar compounds. Recent research from the same authors includes a study on the recovery of phenolic antioxidants from grape stems [8].

### 2.3. Drugs

Another work conducted by Ibrahim et al. and included in this SI comprehensively examines the protective effects of methylene blue (MB) against cardiotoxicity induced by doxorubicin (Dox) [contribution 6], a commonly used chemotherapeutic drug that is known for its efficacy in cancer treatment [9] but is associated with cardiotoxic side effects, leading to heart failure [10]. The mechanism of Dox-induced cardiotoxicity is complex and involves oxidative stress, apoptosis, mitochondrial dysfunction, calcium dysregulation, iron overload, and the disruption of cellular antioxidant defense systems [11,12]. In turn, MB, a phenothiazine derivative, has remarkable antioxidant properties that play a crucial role in cell protection. As an antioxidant, it plays a part in cellular defense systems against oxidative stress [13]. Its anti-oxidative properties [13] and anti-apoptotic effects [14] make it a subject of investigations into its cardioprotective potential. In light of this, Ibrahim’s research group [contribution 6] explored how MB can modulate key pathways involved in cellular defense mechanisms against Dox-induced damage. More precisely, the authors used a rat model to investigate the effect of the administration of MB (4 mg/kg/day, p.o., for 7 days) and Dox (15 mg/kg, i.p.), separately and in combination, on oxidative stress markers, i.e., Kelch-like ECH-associated protein 1 (Keap1), nuclear factor erythroid 2-related factor 2 (Nrf2), glutathione peroxidase 4 (GPx-4), 8-hydroxy-2′-deoxyguanosine (8-OHdG), neurohormonal indicators (noradrenaline), cardiac injury biomarkers (troponin I), and apoptotic mediators (p53 and caspase-3). They found that the Dox administration increased serum troponin I and noradrenaline levels, elevated cardiac Keap1 and the oxidative DNA damage marker (8-OHdG), reduced NFE2L2, Nrf2, and GPx-4 expression, and upregulated p53 and caspase-3. In turn, the co-treatment with MB reduced troponin I and noradrenaline levels, restored Keap1/NFE2L2 (Nrf2)/GPx-4 pathway balance, decreased oxidative DNA damage, and attenuated p53 and caspase-3 activation. Thus, the study indicated that MB can mitigate the cardiotoxic effects of Dox through modulation of the Keap1/Nrf2/GPx4 pathway and caspase-3 activity. These results support further examinations of MB as a potential adjuvant therapy protecting against chemotherapy-induced cardiotoxicity. In another recent study, this research group investigated the potential effect of etoricoxib in reducing inflammation in methotrexate-induced pulmonary injury in rats [15].

### 2.4. Extracts of Plant Origin

The last paper included in this SI focuses on the identification of key active constituents in Eucommia ulmoides Oliv. leaves (EUOL) and the pharmacological mechanisms of EUOL against Parkinson’s disease (PD) [contribution 7], which is a chronic progressive neurodegenerative disorder that primarily affects brain areas responsible for controlling movements [16]. Using a zebrafish PD model, the authors proposed a mechanism for the protective effect of EUOL on neurodegenerative alterations in PD and a site for therapeutic intervention. They found that the 30% ethanol fraction extract of EUOL relieved MPTP-induced locomotor impairments, increased the length of dopaminergic neurons, inhibited the loss of neuronal vasculature, and regulated the misexpression of autophagy-related genes (α-syn, lc3b, p62, and atg7), suggesting that EUOL could serve as a promising candidate for supporting PD treatment. Moreover, they revealed that the 30% fraction extract exerted anti-PD activity by activating 4E-BP1 (the core target protein) and indicated that cryptochlorogenic acid, chlorogenic acid, asperulosidic acid, and caffeic acid were the main components of the 30% fraction extract. On the basis of these results, the authors emphasized the potential of EUOL as a novel functional food or adjunct therapy for PD and concluded that EUOL upregulated 4E-BP1 to inhibit the synthesis of abnormal proteins, thus relieving PD symptoms.

## 3. Conclusions and Outlook

Some of the studies described in this SI provide valuable information about Mg and its potential benefits in the prevention and treatment of such neurodegenerative disorders as AD, PD, and ALS. Others investigate metal-based complexes examined for their potential therapeutic use in AD and in the modern-age disease of cancer. Some studies provide information on how methylene blue can modulate key pathways involved in cellular defense mechanisms against doxorubicin-induced cardiac injury and others highlight the potential of extract from *Eucommia ulmoides* Oliv. leaves as an adjunct therapy for PD, laying a foundation for its future development and application in supporting PD treatment. The results included in this SI also reveal the need for further experimental studies in in vitro and in vivo models to elucidate the biological effects of iron complexes containing differently protonated pyridoxal-S-methyl-isothiosemicarbazone as ligands that are able to bind with DNA and transport proteins. Simultaneously, they emphasize the need to better understand the relationships between the structure and properties of metal complexes, as this may help in efforts to make them therapeutically effective. To summarize, the studies included in this SI may lay the groundwork for future research on new therapeutics that may help to ensure better treatment outcomes in modern-age diseases.

We believe that the information provided in this SI will stimulate the interest of readers in the field of metals and metal-containing complexes with respect to neurodegenerative conditions, such as AD, PD, and ALS, and in the use of metal complexes in cancer treatment. We also believe that this SI will be valuable to readers who are interested in iron complexes with differently protonated pyridoxal-S-methyl-isothiosemicarbazone as ligands in relation to their interaction with key macromolecules, such as DNA and transport proteins, and the potential application of these complexes in biomedical fields. Furthermore, we think that this SI will be of relevance to those interested in the mechanisms of doxorubicin-induced cardiotoxicity and agents with potential cardioprotective properties, as well as to those focused on active constituents from *Eucommia ulmoides* in terms of supporting PD treatment and underlying mechanisms.
